# Report on Late Toxicity in Head-and-Neck Tumor Patients with Long Term Survival after Radiochemotherapy

**DOI:** 10.3390/cancers13174292

**Published:** 2021-08-26

**Authors:** Anna Maria Stefanie Buchberger, Elmar Anton Strzelczyk, Barbara Wollenberg, Stephanie Elisabeth Combs, Anja Pickhard, Steffi Ulrike Pigorsch

**Affiliations:** 1 Department of Ear, Nose and Throat Head and Neck Surgery, Klinikum Rechts der Isar, Technical University of Munich, Ismaninger Str. 22, 81675 Munich, Germany; elmarstrzelczyk@sublimd.com (E.A.S.); barbara.wollenberg@tum.de (B.W.); anja.pickhard@tum.de (A.P.); 2 Department of Radiooncology and Radiotherapy, Klinikum Rechts der Isar, Technical University of Munich, Ismaninger Str. 22, 81675 Munich, Germany; stephanie.combs@tum.de (S.E.C.); steffi.pigorsch@tum.de (S.U.P.)

**Keywords:** late toxicity, HNSCC, head-and-neck tumor patient, long-term survival, radio-(chemo-)therapy, late-onset radiotoxicity

## Abstract

**Simple Summary:**

Regular tumor follow-up care provided by ear-nose-throat specialists ends when patients reach 5-year survival, but radio-toxicity is a lifelong process. In this study, long-term head-and-neck cancer survivors undergoing tumor FU-care exceeding five years were analyzed for late onset symptoms after radio-(chemo-)therapy. Almost one third of these patients developed new radiation associated symptoms beyond the common 5-year tumor follow-up margin. Previous radiotherapy led to a two-fold increase for late-onset new complaints, especially after irradiation of the lymphatic pathways in the neck. These findings underline the need for a life-long tumor-follow-up care for long-term head-and-neck cancer survivors.

**Abstract:**

Regular tumor follow-up care provided by ear-nose-throat (ENT) specialists ends when patients reach 5-year survival, but radiotoxicity is a continuous lifelong process. In this study, long-term head-and-neck cancer (HNC) survivors undergoing tumor follow-up (FU) care exceeding five years in a certified HNC center of a German university hospital were analyzed for newly diagnosed late sequelae after radio-(chemo-)therapy. Patients diagnosed with squamous cell carcinoma (SCC) of the oral cavity, larynx or oro-/hypopharynx receiving treatment between 1990 and 2010 with a tumor FU care beyond five years were reviewed retrospectively for signs of late sequelae after radio-(chemo-)therapy (R(C)T) including carotid artery stenosis, stenosis of the cranial esophagus, dysphagia, osteoradionecrosis, and secondary malignancies. Long-term survivors that solely received surgical treatment served as control. Of 1143 analyzed patients we identified 407 patients with an overall survival beyond five years, 311 with R(C)T and 96 patients without R(C)T. Furthermore, 221/1143 patients were lost to FU and the mortality rate within the first 5-years was 45%. Moreover, 27.7% of the long-term survivors were diagnosed with new onset late sequelae within the following five years. RT was significantly associated with a two-fold risk increase for newly diagnosed symptoms, especially after RT of the lymphatic pathways (LP) which showed a hazard ratio of 23.3 to develop alterations on the carotid arteries. Additional chemotherapy had no statistical correlation with any late onset toxicity nor did the mode of R(C)T (adjuvant/definitive). Although the validity of this study might be limited due to its retrospective nature and the dependence on the voluntary participation in a prolonged tumor FU, the results nevertheless provide the need to offer and encourage a tumor FU by ENT specialists exceeding the common 5-year margin. This could prevent secondary morbidities and improve quality of life for long-term cancer survivors.

## 1. Introduction

Side effects of radiation therapy (RT) are categorized into acute and late toxicities. The former describes damages occurring within the first day of RT until day 90. Late or chronic radiation induced side effects develop after day 90 and last lifelong [1]. This definition was harmonized and stated by the European Organization for Research and Treatment of Cancer (EORTC) as well as the Radiation Therapy Oncology Group (RTOG), which implemented the LENT-SOMA scale (Late Effects of Normal Tissues-Subjective Objective Management Analytic) to evaluate short- and long-term therapy effects [2,3]. Fibrosis, atrophy, vessel injury, infertility, hormonal disturbances and secondary cancer are categorized as late sequelae of therapy [4,5].

Ionizing radiation primarily induces single- and double-strand breaks of the deoxyribonucleic acid (DNA) and changes the cells’ microenvironment through reactive oxygen species leading to partial apoptosis [6]. Hence, rapidly proliferating tissues (e.g., skin, mucosa) react with acute inflammation. In tissues with low cellular turnover, DNA-damage and changes in the microenvironment also occur, but these processes are not dominated by cell division. They are rather based on chemokines and inflammatory as well as fibrotic cytokines and subsequently change intercellular interaction and cellular migration. Thus, there is a latency between radiation and the occurrence of tissue damage such as tissue fibrosis, tissue atrophy or vascular injury [7]. This entire process is similar to the process of chronic healing. After an initial inflammation these cytokines trigger a change in the recruitment of fibroblasts which differentiate to collagen producing fibrocytes, increasing the extracellular matrix production leading to a progressive stiffness of the tissue. In addition to the processes of inflammation and fibrosis, thrombotic and ischemic events occur [8].

To date, no correlation between the severity of acute reactions to radiotoxicity such as radiodermatitis and radiomucositis and late onset radiotoxicities like fibrotic damage has been found [9]. The inter-individual different reactions to radiation therapy is explained by varying genotypes of genes which are involved in the detection and repair of DNA damage, in the inflammation and signaling pathways, apoptosis and proliferation [4].

The most important risk factor for the occurrence of acute and late radiation induced complications after treatment of the head-and-neck region are the single and total radiation dose applied and the radiosensitivity of the affected tissue [10,11,12]. Due to novel radiation techniques like the intensity modulated radiation therapy (IMRT), side effects are greatly reduced compared to conventional radiotherapy [12] since neighboring sensitive structures can be more protected [13,14,15]. Further risk factors for acute complications are female gender, low Karnofsky index, high body-mass-index and advanced tumor stage [16]. Risk factors for late complications after RT are advanced age, high T-status, primary tumors of the larynx and hypopharynx, female gender and a pronounced weight loss during RT [5,11,16]. There are also predisposing genetic and ethnic factors that influence the occurrence of late-onset complications. This seems plausible based on the predicted similarities in the explanation models of wound healing and late toxicity [17].

Since late sequelae after RT develop in a dynamic and progressive process, first manifestations of late radiogenic damages can occur lifelong.

For ENT specialists in Germany, the regular tumor follow-up care for head-and-neck cancer patients ends after five years. As pointed out, complications after definitive or adjuvant radio-(chemo-)therapy can appear after this time period and severely influence the patients’ morbidity and quality of life. Thus, radio-oncologists already reached a consensus on offering lifelong follow-up care to their patients.

The aim of this retrospective study was to analyze the late occurrence of new complaints associated with radiation treatment in long-term head-and-neck cancer survivors later than five years after the end of RT. We aim to demonstrate and underline the need for a lifelong tumor follow-up by a multidisciplinary team of ENT specialists and radio-oncologists.

## 2. Patients and Methods

We retrospectively analyzed a large collective of head and neck squamous cell carcinoma (HNSCC) patients with tumors of the oral cavity, oropharynx, hypopharynx and larynx of a certified head-and-neck cancer center of a university hospital in Germany. Included were all patients who were diagnosed between 1990 and 2010 with the following ICD-10 (International Statistical Classification of Diseases and Related Health Problems) codes: C01-06, C09, 10, 12, 13, 14, 32, 77.0 and 80 and a follow-up beyond five years who had undergone surgery without (control-group) or with adjuvant R(C)T or received definitive R(C)T (R(C)T = study-group). Patient documentation was searched including paper charts from both departments for ENT, Head-and-Neck Surgery and Radio Oncology as well as the clinic’s computer-based information system SAP (SAP ERP 6.0, IS-H, SAP SE Walldorf, Germany). This study was approved by the ethics committee of the Medical Faculty of the Technical University of Munich (No.: 111/16S).

Included were all patients with a follow-up period exceeding five years. All available sources of information were systematically searched for late toxicities/signs of late sequelae with a new diagnosis beyond the five-year margin. Diagnoses included were carotid artery stenosis, changes of the carotid arteries diagnosed by the center of vascular surgery, secondary/metachronic carcinomas in the head-and-neck region, stenosis of the cranial third of the esophagus, osteoradionecrosis, new onset of difficulties with the tracheo-esophageal fistula after laryngectomy, dysphagia and others (accessory syndrome, cerebral venous sinus thrombosis, cholesteatoma in the radiation field).

## 3. Statistics

Statistical analysis was performed using the “statistical package for social sciences” (IBM SPSS Statistics, version 24.0.0.0, IBM Inc., Chicago, IL, USA). A descriptive analysis for absolute and relative frequencies including the mean and standard deviation for the study collective was performed. The last day of the regular five-year tumor follow-up care was set as time point “0”, the maximal follow-up time was limited to ten years and only new events occurring in this time period were included in the following calculations. The reason for this time restriction was to avoid the bias of complaint-driven doctor visits. Beyond the 10-year border we found a massive drop of follow-up visits and a suspiciously high number of complaint-driven visits. Kaplan–Meier curves for the occurrence of late sequelae after radiation therapy were generated, lost-to-follow-up and death were censored. The impact of RT on the occurrence of late sequelae per se comparing the groups RT/RCT vs. no RT was calculated using the log-rank and chi-square test. To calculate the risk (hazard ratio) of experiencing a late sequela due to previous RT we used Cox regression. Additionally, we evaluated the influence of RT for the individual late onset complaint with log-rank and chi-square tests. We tested for effects of the following parameters within the therapy-subgroup of patients with primary or adjuvant RT for the appearance of late sequelae after five years: gender, age, chemotherapy, surgery (especially neck dissection), RT of the lymphatic pathways as well as alcohol and tobacco abuse.

## 4. Results

### 4.1. Patient Characteristics

**Figure 1:**
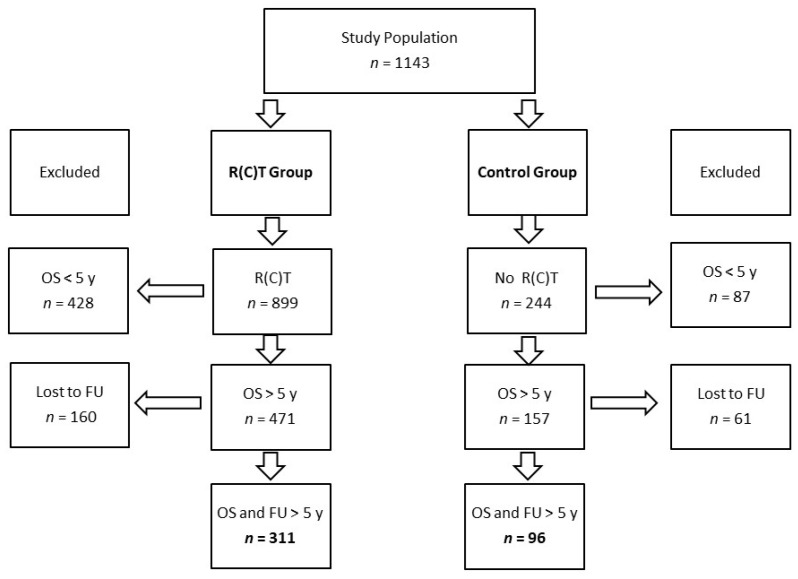
CONSORT diagram. Abbreviations: R(C)T = radio(chemo)-therapy, OS = overall survival, FU = follow-up, y = years.

We identified 1143 patients with head-and-neck squamous cell carcinoma (HNSCC) of the oral cavity, larynx, oro-and hypopharynx who were diagnosed and treated at the Department for ENT and most of them at the Department of Radio Oncology and Radiotherapy of the Technical University of Munich from 1990 to 2010. The CONSORT diagram shows the group sizes of the study population (Figure 1).

The patients’ characteristics of the RT group (*n* = 311) and the control group (*n* = 96) of long-term survivors is summarized in Table 1.

The 5-year overall survival of the RT group was 34.6% (311/899) and 39.3% (96/244) in the control group. The 10-year overall survival was 7.8% for both groups (70/899; 19/244) (Table 1).

### 4.2. Late Radiation Induced Toxicities

Newly diagnosed late complaints beyond the five-year margin after initial tumor diagnosis were found in 86 (27.7%) of the irradiated patients, respectively 72 patients (23.2%) within the 10-year follow up margin (Table 2). In the control group we found 13 patients (13.5%), respectively 11 patients (11.4%) within the 10-year follow up margin, with new, similar complaints which were clearly not radiation associated.

The newly diagnosed complaints are illustrated in Table 2. Carotid artery stenosis was found in 22.2% (16/72), alterations of the carotid arteries in 13.9% (10/72) and metachronic carcinomas in 11.1% (8/72) in the RT group within the 10-year follow up. In the non-RT group stenosis of the carotid arteries was found profoundly less often, in 9.1% (1/11 patients), whereas changes in the carotid arteries was documented more often, in 27.3% (3/11). Metachronic carcinomas were found in 45.5% (5/11) within the 10-year follow up. For both groups, more than 60% of patients with metachronic carcinomas had documented nicotine consumption (RT-group 5/8 and non-RT group 3/5). The average time for diagnosis of a new complaint was 7.1 years (SD ± 1.3) for RT patients and 7.3 years (SD ± 1.4) in the control group. However, metachronic carcinomas were mostly detected significantly later and were one reason for complaint-driven visits beyond the 10-year margin (8/72 RT-patients within the 10-year margin vs. 7/14 RT-patients beyond the 10-year margin; 5/11 control-patients within the 10-year margin vs. 2/2 patients beyond the 10-year margin. Beyond the 10-year margin, all patients with metachronic carcinomas in both groups had a documented history of nicotine consumption. The average time of diagnosis for metachronic carcinomas was at 12.78 years (7.13–18.46 years).

**Figure 2:**
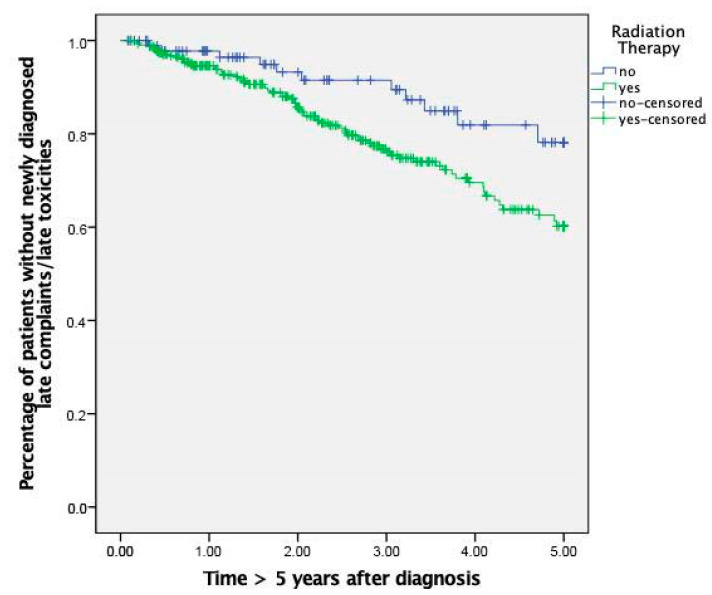
Kaplan–Meier curve displaying the difference in the incidence of late complaints between RT-group and control group.

The number of late sequelae was significantly higher in the RT group compared to the control group (*p* = 0.02) (Figure 2).

The hazard ratio was 2.07 (95%-CI 1.10–3.91; *p* = 0.03), thus, radiation leads to a twofold increase in the risk to exhibit a new late complaint beyond the five-year margin. The RT group showed a trend to be diagnosed more often with a stenosis of the carotid arteries (*p* = 0.09) and second carcinomas (*p* = 0.17), esophageal stenosis (*p* = 0.18) and osteoradionecrosis (ORN) (*p* = 0.07). Insufficiencies of the vocal prosthesis tracheo-esophageal fistula (*p* = 0.09) was exclusively found in the RT group. The incidence of dysphagia as a new complaint was similar in both study groups (*p* = 0.73).

Between the four different radiotherapy groups (adjuvant RT, adjuvant RCT, definitive RT und definitive RCT) there was no difference in the frequency of late sequelae (*p* = 0.78). Concurrent chemotherapy (always including cisplatin, 20 mg days 1–5 and days 29–32, afterwards 40 mg weekly) did not have a statistically relevant impact on the number of complaints detected (*p* = 0.33) nor did the mode of RCT in consideration of definitive (70 gy) or adjuvant treatment (60–64 gy) (*p* = 0.63) for head and neck cancer or if surgery was performed (*p* = 0.63).

**Figure 3:**
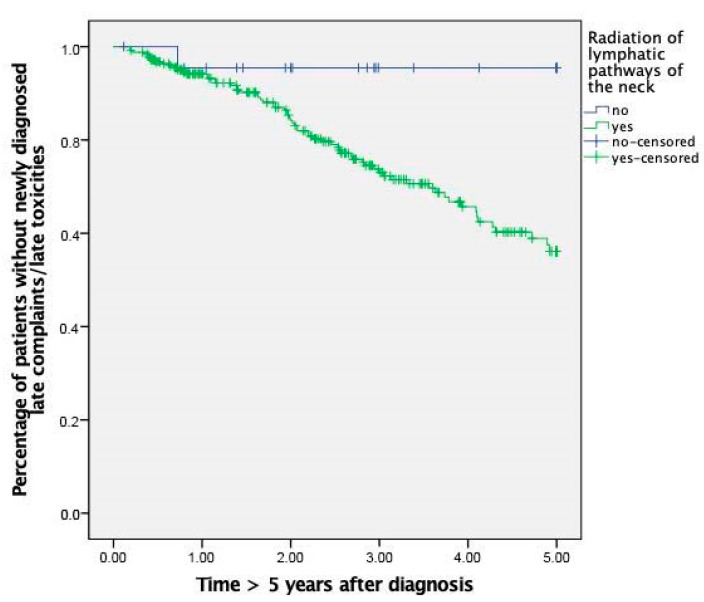
Kaplan–Meier curve displaying the effect of radiation therapy of the lymphatic pathways of the neck on late complaints.

After RT of the lymphatic pathways of the neck region, late complaints were generally diagnosed significantly more often (*p* = 0.02) with a hazard ratio of 7.16 (95%-CI 0.99–51.56; *p* = 0.05) (Figure 3), without reaching significance for any specific type of late toxicity as mentioned above. The most profound trend towards a correlation was found between RT of the LP and a damage of the carotid arteries (stenosis and alterations) (*p* = 0.11) with a hazard ratio to develop stenosis or alterations of the carotid arteries after irradiation of the neck region of 23.31 (95%-CI 0.06–9051; *p* = 0.30).

When split into subgroups based on tumor location, differences in the type of late sequelae in the study group as well as control group were observed in absolute numbers. Patients of the study group diagnosed with HNSCC of the oropharynx showed the highest number of late sequelae of the carotid arteries as well as osteoradionecrosis, while patients after carcinomas of the oral cavity showed the highest number of second carcinomas. In the control group, second carcinomas were most frequently found in patients with laryngeal carcinomas while alterations of the carotid artery were most often observed in patients with carcinomas of the oral cavity.

Gender did not show a significant influence to experience new late complaints (*p* = 0.47) for both groups, nor did prior/ongoing abuse of alcohol or tobacco (*p* = 0.83; *p* = 0.77) for the study group. For the control group however, vascular changes (carotid artery stenosis or alteration) where significantly associated with tobacco consumption (*p* < 0.05). For age, we found a 10.5% increase in the risk of experiencing new late sequelae with each age gain of 10 years, which was statistically not significant (*p* = 0.415).

## 5. Discussion

The standard time period for tumor follow-up care for patients diagnosed with HNSCC performed by ENT specialists is five years [18]. National guidelines for radiation oncology advise a lifelong tumor and side-effect follow up after radiotherapy of any kind for malignancies in general [19].

Our data emphasizes the significance of RT as a possible cause of late sequelae after tumor therapy in head-and-neck cancer. Patients who had undergone RT were diagnosed with late complaints beyond the five-year margin twice as often as patients that had only been treated with surgery. Within the group of irradiated patients, especially those after RT of the lymphatic pathways were significantly affected. For the individual complaints, we were only able to show a trend towards a correlation with RT, due to the small group sizes. However, it has previously been shown that RT of the lymphatic pathways leads to radiation induced pathologies of the carotid arteries [20,21,22] for which we found a trend. The group of irradiated patients exclusively suffered from stenosis of the proximal esophagus, an association which has previously been established with RT [5,11,23,24,25] as well as problems with the tracheo-esophageal fistula for the vocal prosthesis after laryngectomy. These two late complaints, as well as osteoradionecrosis which is per definition caused by RT [26], were diagnosed rather late and notably later than five years after treatment. Due to the small number of patients and cases diagnosed, these complaints failed to reach statistical significance. However, since almost one third of irradiated HNSCC patients were affected by new complaints possibly related to the RT on average seven years after the initial diagnosis, our data emphasizes the need to provide an extended tumor follow-up care beyond the standard five years.

The collective was divided into an intervention group with irradiated patients and a control group with only surgical therapy. Although age and gender were comparable between the subgroups, they differed significantly with regards to TNM status, grading, UICC tumor stadium and location of the primary tumor. While irradiated patients were diagnosed with a locally advanced HNSCC UICC-stage IVA in 53.7%, control patients were found to be in limited UICC-stage I in 74%. Differences in the groups are explained by the nature of HNSCCs and their therapy options advised by current guidelines, for example, adjuvant or definitive radiation therapy for patients with lymph node metastasis and locally advanced primary tumors [18,27]. The small number of patients with high TNM status within the control group are explained by patients’ refusal to receive RT or intercurrent complications with the need to end RT early.

The higher overall as well as the prolonged medium survival of the control group is owed to the limited stage of the tumor. Five-year overall survival was thus 39.3% in the control group compared to 34.6% of the RT group. However, interestingly the ten-year overall survival was identical for both groups with 7.8%.

The differences in baseline characteristics (mainly tumor stage) between the RT and control group are caused by treatment guidelines concerning the different tumor stages as well as the retrospective nature of this study and limit its power. Nevertheless, the number of newly diagnosed complaints, influencing patients’ morbidity and quality of life is high and emphasizes the need for a lifelong tumor follow-up care by a multidisciplinary caretaker team.

The long timeframe of tumor follow-up care analyzed is another limitation of this study. We found a certain level of inhomogeneity in the quality of data in areas such as documentation quality in general (varying doctors saw the patients, LENT-SOMA scale had not been implemented yet for years), mode of RT offered (IMRT was introduced in 2008 in our hospital), poorer standards of follow-up imaging and sensibility to the issue of late radiotoxicity in general.

We limited the maximum follow-up time for the statistics for new onset late sequelae after tumor therapy to ten years. Since there is no official recommendation to provide tumor follow-up care beyond the standard five-year margins, we suspected an increasing number of complaint-driven doctor consultations of long-term HNSCC survivors which could bias the collective. The same might be true for patients presenting beyond the five-year margin one could argue, but our clinic has propagated prolonged tumor follow-up for decades. Additionally, newly diagnosed complaints were detected “only” in approximately 30% of the visits. Even with a possible bias this should nevertheless not detract from the necessity and impact of this analysis.

The high number of patients lost to follow-up (17.8% for RT-patients (160/899), 25.6% for patients of the control group (61/238)) is owed to the retrospective nature of this analysis and the lack of standardized guidelines advising a lifelong tumor follow-up for ENT specialists. Additionally, it supports the call for a central tumor registry [28].

A fundamental weakness of a retrospective analysis is the question of causality. Complaints and late sequelae that have been subsumed as late toxicities could also have been influenced or caused by other risk factors [29,30]. This is, for example, the case for the diagnosis of second or metachronic carcinomas in this analysis. They might have been induced by RT, but since there is a significant number of metachronic carcinomas of the head and neck region in the control group, i.e., the influence of consumed noxae or ongoing addiction to alcohol and tobacco also has to be considered and was documented in over 60% of both patient groups within the 10-year margin and 100% of all patients diagnosed with metachronic carcinomas beyond the 10-year margin. In the current literature a causality for secondary malignancies is postulated in case of a diagnosis ten years after radiotherapy [31,32,33], but seems to make up only 8% of diagnosed metachronic carcinomas [34]. In fact, we found 15 metachronic carcinomas in the RT group with almost 50% beyond the ten-year margin (Table 2). However, even if the causality of secondary carcinomas and RT cannot be proven with this set of data, our numbers support the need for a lifelong tumor follow-up care since a significant number of patients with and without previous RT for HNSCC were diagnosed with metachronic carcinomas. Because previous therapies (e.g., structural defects due to previous surgery) might narrow down subsequent treatment options, an early detection during follow-up examination could have a huge benefit for the patient. Furthermore, previous and ongoing consumption of noxious agents such as alcohol and nicotine that were distributed equally increase the risk of metachronic carcinoma and justify an ongoing follow-up for every HNSCC patient.

The question whether or not high age is a risk factor for late toxicities is not explicitly answered in literature. Machtay et al. [11] did correlate age with late toxicities whereas Meyer et al. [16] did not. In our calculations, we found a risk increase of 10.5% per decade age gain but this result was not statistically significant.

Additionally, definitive or adjuvant chemotherapy failed to impact late toxicities significantly, as shown previously in literature [35].

The awareness of radiation induced late toxicities in long-term head-and-neck cancer survivors has improved significantly in the last decade. It was advised to provide a prolonged tumor follow-up care since patients share many comorbidities such as cardiovascular pathologies or increased risk for metachronic tumors due to ongoing abuse of associated noxious agents such as alcohol and tobacco [36]. With a prolonged or possibly lifelong tumor follow-up, late toxicities, late complaints as well as therapy or noxae associated comorbidities could be detected earlier and thus possibly increase overall survival and quality of life, as has been shown for other tumor etiologies [37,38,39].

## 6. Conclusions

Previous radiation therapy, regardless of its mode (definitive or adjuvant) or the addition of chemotherapy leads to a two-fold risk increase for the development of late-onset irradiation associated complaints in head-and-neck cancer long-term survivors. Almost one third of these long-term survivors experience late toxicities beyond the five-year survival margin. However, complaints were also found in the non-irradiated control group, although to a lesser extent, possibly based on the therapy or shared abuse of associated noxious agents such as alcohol and tobacco. Thus, our data strongly support the need to provide all head-and-neck cancer patients with a prolonged tumor follow-up care exceeding the common five-year survival margin. With this extended or possibly lifelong tumor follow-up, late toxicities, late onset new tumortherapy-associated complaints as well as noxae-associated comorbidities will be detected timely and could thus possibly either increase overall survival, quality of life, or in combination.

## Figures and Tables

**Table 1:** Patient characteristics.

Category	Feature	RT		Control	
		*n*	%	*n*	%
Gender	Male	239	76.8	77	80.2
	Female	72	23.2	19	19.8
	Total	311	100	96	100
Age at	Median (range)	57.7 (27.8–88.8)		61.3 (39.0–90.3)	
Diagnosis (years)	Mean ± SD	57.6 ± 9.8		62.1 ± 10.1	
Localization of Primary Tumor	Oral cavity	38	12.2	25	26
	Oropharynx	146	46.9	18	18.8
	Hypopharynx	52	16.7	1	1
	Larynx	63	20.3	52	54.2
	≥2 synchronic tumors	9	2.9	0	0
	Cervical CUP	3	1	0	0
	Total	311	100	96	100
T-Stadium	1	83	26.7	76	79.1
	2	97	31.2	14	14.6
	3	68	21.9	3	3.1
	4	60	19.2	1	1
	n/a	3	1	2	2.1
	Total	311	100	96	100
N-Stadium	0	105	33.8	73	76
	1	56	18	3	3.1
	2a	18	5.8	3	3.1
	2b	81	26	4	4.2
	2c	35	11.3	1	1
	3	8	2.6	0	0
	n/a	8	2.6	12	12.5
	Total	311	100	96	100
M-Stadium	0	246	79.1	66	68.8
	1	2	0.6	0	0
	X	51	16.4	28	29.2
	n/a	12	3.9	2	2.1
	Total	311	100	96	100
Grading	1	17	5.5	9	9.4
	2	141	45.3	66	68.8
	3	144	46.3	20	20.8
	4	2	0.6	0	0
	n/a	7	2.3	1	1
	Total	311	100	96	100
UICC-Stadium	I	23	7.4	71	74
	II	40	12.9	11	11.5
	III	79	25.4	4	4.2
	IVA	155	49.8	8	8.3
	IVB	11	3.5	0	0
	IVC	2	0.6	0	0
	n/a	1	0.3	2	2.1
	Total	311	100	96	100
Therapy	No Radiation	0	0	96	100
	Definitive RT	15	4.8		
	Definitive RCT	58	18.6		
	Adj. RT	77	24.8		
	Adj. RCT	161	51.8		
	Total	311	100		
Risk factors	Yes	194	62.4	60	62.5
Tobacco	No	54	17.4	3	3.1
	n/a	63	20.3	33	34.4
	Total	311	100	96	100
	Yes	134	43.1	31	33.3
Alcohol	No	114	36.7	32	32.3
	n/a	63	20.3	33	34.4
	Total	311	100	96	100
Follow-Up (years)	Median (range)	8 (5.1–25.1)		8.1 (5.1–14.6)	
	Mean ± SD	8.5 ± 2.8		8.1 ± 2.3	
Radiation (total dose of RT (Gy)	Number of Pat.	250 *			
	Median Gy (range)	64 (34.0–78.0)			
	Mean Gy ± SD	63.8 ± 5.8			

Abbreviations: *n* = number, RT: radiation therapy, RCT: radiochemotherapy, Co: control, FU: follow-up, SD: standard deviation; Gy: (gray) unit for dose of radiotherapy, * = 61 patients were radiated elsewhere and documentation about the applied radiation dose could not be retrieved. Note: a differentiation between ongoing and prior tobacco/alcohol abuse nor the exact amount of consumption was possible due to the retrospective nature of the study.

**Table 2:** Overall survival and diagnosed-late complaints/late toxicities beyond five-year margin.

Variation		RT		Control	
		*n*	%	*n*	%
OS	5-y OS	311	34.6	96	39.3
	10-y OS	70	7.8	19	7.8
Late Complaints	Total	86	27.7	13	13.5
	FU for 10 years	72	23.2	11	11.4
Complaints Diagnosed during entire FU	Stenosis of Carotid Artery	21	24.4	1	7.7
	Changes of Carotid Artery	11	12.8	3	23.1
	Metachronic Carcinoma	15	17.4	7	53.8
	Stenosis of prox. Esophagus	6	7	0	0
	Osteoradionecrosis	11	12.8	0	0
	Problems with Tracheo-esophageal Fistula	11	12.8	0	0
	Dysphagia	9	10.5	2	15.4
	Others	2	2.3	0	0
	Total	86	100	13	100
Complaints Diagnosed with max. 10-y FU	Stenosis of Carotid Artery	16	22.2	1	9.1
	Changes of Carotid Artery	10	13.9	3	27.3
	Metachronic Carcinoma	8	11.1	5	45.5
	Stenosis of prox. Esophagus	6	8.3	0	0
	Osteoradionecrosis	11	15.3	0	0
	Problems with Tracheo-esophageal Fistula	10	13.9	0	0
	Dysphagia	9	12.5	2	18.2
	Others	2	2.8	0	0
	Total	72	100	11	100
Time to new Complaint (years)	Number of Patients	86		13	
	Median (range)	7.2 (5.2–20.8)		8.1 (5.3–11.1)	
	Mean ± SD	8.2 ± 2.9		7.9 ± 1.9	
Time to new Complaint with FU max. 10 years	Number of Patients	72		11	
	Median (range)	7.0 (5.2–9.9)		7.0 (5.3–9.7)	
	Mean ± SD	7.1 ± 1.3		7.3 ± 1.4	

Abbreviations: *n* = number, RT: radiation therapy, OS: overall survival, FU: follow-up, SD: standard deviation.

## Data Availability

The original data analyzed in this study can be obtained from the corresponding author upon reasonable request.
